# Modelers of students’ entrepreneurial intention during the COVID-19 pandemic and post-pandemic times: The role of entrepreneurial university environment

**DOI:** 10.3389/fpsyg.2022.976675

**Published:** 2022-09-06

**Authors:** Simona Mihaela Trif, Gratiela Georgiana Noja, Mirela Cristea, Cosmin Enache, Otniel Didraga

**Affiliations:** ^1^Department of Marketing and International Economic Relations, Faculty of Economics and Business Administration, West University of Timişoara, Timişoara, Romania; ^2^Department of Finance, Banking and Economic Analysis, Center for Economic, Banking and Financial Research (CEBAFI), Faculty of Economics and Business Administration, University of Craiova, Craiova, Romania; ^3^Department of Finance, Faculty of Economics and Business Administration, West University of Timişoara, Timişoara, Romania; ^4^Department of Business Information Systems, Faculty of Economics and Business Administration, West University of Timişoara, Timişoara, Romania

**Keywords:** entrepreneurial intentions, entrepreneurship education, students’ innovativeness, COVID-19, structural equation modeling, Gaussian graphical models

## Abstract

This paper examines the shaping factors, drivers, and impact credentials of students’ entrepreneurial intention during the COVID-19 pandemic. The proposed framework addresses the antecedents of entrepreneurial intention among students in Romania, focusing on three focal constructs, namely, risk-taking, proactiveness, and innovativeness, with a keen focus on the mediation effect of the entrepreneurial university environment. The study used self-reported data collected through an online questionnaire during November 2020–February 2021 from a sample of 1,411 students in western Romania. The methodology relies on two modern techniques of modeling cross-sectional data, namely, structural equation modeling (SEM) and Gaussian graphical models (GGMs). The main results highlight that the three constructs positively relate to students’ entrepreneurial intention in a comprehensive framework where the entrepreneurial university environment drives innovativeness. The paper brings forward, in an innovative way, that entrepreneurship education and training at the university level enhance students’ entrepreneurial intentions as it fosters the attainment of advanced knowledge and skills. The results are well associated with the start-up process as prerequisites for successful entrepreneurship engagement of youth in a globalized digital economy, particularly during this challenging pandemic outbreak, but also post-pandemic times. This research sheds new light on the essential role played by higher education institutions in providing advanced knowledge and necessary skills matched with the labor market needs, thus enhancing students’ innovativeness and their entrepreneurial intentions.

## Introduction

The year 2019 will be remembered as the year when the coronavirus disease (COVID-19) started to spread globally, impacting both developed and developing economies, with severe negative consequences on people’s health and wellbeing, employees’ resilience, and a ravaging impact on the business sector (Luches et al., 2021; Mihalca et al., 2021; Tonkin and Whitaker, 2021; Luches et al., 2022; Meyer et al., 2022).

Countries worldwide were affected by the pandemic, and the many lockdowns have led to a crisis in all sectors of economic activity, with a severe impact on the labor market. Mihalca et al. (2021) highlight that the COVID-19 pandemic brought unprecedented challenges for employees in terms of their job security, working conditions, productivity, and the nature of their work. According to Fleming (2021), the pandemic has damaged particularly young employees due to unstable economic conditions, young people, particularly young women, facing higher unemployment levels than adults, which continue to accelerate faster. A possible solution to reduce unemployment is to encourage young people to indulge in entrepreneurial activities, in synergy with the acknowledgment of innovation and “futuristic thinking” that “can turn the hardships caused by the pandemic into opportunities” (Ratten, 2021, p. 2). Therefore, it is important to turn this negative period into a positive one and to change public perceptions about the pandemic crisis. Entrepreneurship, therefore, becomes requisite in times of crisis as it lays out a positive perspective on the new conditions (Ngugi and Goosen, 2021). Withal, young highly educated individuals are more oriented to entrepreneurial intentions (EIs) and actions than adult people with or without advanced education (Turker and Sonmez Selcuk, 2009). Despite the utmost significance of the topic, there is a general gap in the literature in understanding the effects of the COVID-19 pandemic on entrepreneurship (Ratten, 2020), especially on the side of young individuals.

In this frame of reference, the current study brings new theoretical groundings and empirical evidence to address the awareness of the entrepreneurial actions for the young highly educated individuals after the coronavirus pandemic time. The proposed framework focuses on identifying the antecedents of EI among students in western Romania during the COVID-19 pandemic. The methodological endeavor is configured through the lens of structural equation modeling (SEM), as a modern and complex technique designed to process cross-sectional data compiled through the maximum likelihood estimator (MLE) method, complemented by Gaussian graphical models (GGMs). The research design captures, in a gradual framework, the direct, indirect, and total interlinkages between three major drivers of EI, namely, risk-taking (RISK), proactiveness (PROACT), and innovativeness (INOV), along with the mediated impact of the entrepreneurial university environment (EUE), and their further cumulated impact on entrepreneurial intention (EI).

Therefore, through its complex and innovative approach, this study entails the shaping factors of entrepreneurial intentions of youths in outstanding circumstances caused by the COVID-19 pandemic and establishes the positive impact of entrepreneurship education and university environment on entrepreneurial intention. The study sheds new light on the essential role played by higher education institutions in providing advanced knowledge and necessary skills matched with the labor market needs and tailored to professional profiles, thus enhancing students’ innovativeness and through it, their entrepreneurial intentions, particularly during this challenging COVID-19 pandemic, but also post-pandemic times. The findings of the current research are consistent with those of the literature, while also providing new insights on key credentials that drive entrepreneurial intention in challenging pandemic and post-pandemic times, considering that there is a dearth of empirical evidence on this topical subject approached in a similar context. The research is innovative also through the methodological endeavor and particularly as besides SEM, it applies another advanced modern econometric technique, namely, the network analysis performed through GGMs, which is less considered in studies approaching similar topics.

The current research has both theoretical and practical implications, rendering global the fundamental importance of entrepreneurial education and training in a competitive integrative framework that blends traditional and modern teaching and learning techniques, overcoming pandemic barriers, and aligning with the opportunities of the globalized digital economy.

The remainder of this paper is configured as follows. First, the theoretical background of the focal constructs is presented, with reference to entrepreneurial intention, individual entrepreneurial orientation (innovativeness, risk-taking, and proactiveness), the synergy between them, and the entrepreneurial university environment. Second, the hypotheses on the precursors of entrepreneurial intention are entailed based on the relevant entrepreneurial literature. The third section encompasses the description of the research methodology and the results of the data analysis. Finally, there is a discussion of the findings of this study, followed by strategies/policies proposed to enhance entrepreneurial higher education, also rendering the limitations of the study and suggestions for further research.

## Theoretical groundings and hypotheses development

### Entrepreneurial intention

Intentionality is known in the literature (Bandura, 2001) as a core feature of human beings. Moreover, intention affects individuals’ choices because it is a rendering of the meaning of future action (Moriano et al., 2012). In addition, intention becomes a basic element in explaining behavior (Linan et al., 2011), as well as in foretelling ways of acting at the individual level (Ajzen, 1991). According to Bird (1988, p. 442), intention “is a state of mind directing a person’s attention toward a specific objective or a path in order to achieve something.”

As regards entrepreneurial intention and starting a new business, in the last years, these features have received much attention from researchers. On this line, the “Theory of Planned Behavior” (TPB) (Ajzen, 1991; Sun et al., 2017) enhances entrepreneurial intention as a prerequisite to entrepreneurial behavior, while certain specific attitudes predict intention. Driven by TPB, the entrepreneurial intention represents an aspiration of individuals to do fruitful activities that direct them to avail themselves of relevant concepts of new business and to effectively implement them (Krueger et al., 2000). Turker and Sonmez Selcuk (2009, p. 146) acknowledged that the entrepreneurial intention of university students arises from their awareness of practicality actions, which are shaped by the university environment, conjunctural factors (social, economic, political or cultural, considered as “structural support”), their “personality trait, self-confidence,” and the degree of support by family and friends (“relational support”). Withal, mentoring on entrepreneurship also has a great impact on the entrepreneurial intention of university students, being proved that their intention was doubled compared with that of the students that did not benefit from specialized entrepreneurial training (Gimmon, 2014).

### Individual entrepreneurial orientation

Entrepreneurial orientation represents the individuals’ propensity for opening a new business (Langkamp Bolton and Lane, 2012). A significant first insight into the concept of entrepreneurial orientation was provided by Miller (1983, p. 771), at the level of firms, that suggested that an entrepreneurial company is one that “engages in product market innovation, undertakes somewhat risky ventures, and is first to come up with “proactive” innovations, beating competitors to the punch.”

Based on this statement, the salient factors for entrepreneurial orientation at the firms’ level are innovativeness, risk-taking, and proactiveness. At the individual level, Langkamp Bolton and Lane (2012) counted on five variables, namely, autonomy, innovativeness, willingness to take risks, proactiveness, and competitiveness, to measure entrepreneurial orientation, as previously proposed by Lumpkin and Dess (1996). Ultimately, after performing the factor analysis as regards the leading variables for entrepreneurial orientation, obtaining 60% of the total variance, three noticeable factors were retained by them (Langkamp Bolton and Lane, 2012), namely, innovativeness, risk-taking, and proactiveness. These credentials are highly agreed upon in the literature (So et al., 2017; Linton, 2019) as the main drivers of the individual entrepreneurial orientation (IEO), each of them representing a distinctive concept (Lyon et al., 2000; Naldi et al., 2007), as further entailed.

Innovativeness is considered a core entrepreneurial personality trait that inspires entrepreneurs (Roy et al., 2017). Wathanakom et al. (2020) asserted that innovativeness is a primary motivation in starting a new business venture, and it is considered an act of creativeness. Innovativeness can be defined from a technological, behavioral, and product-related perspective (Salavou, 2004). From a behavioral perspective, innovativeness indicates behavioral change, and it can be viewed as a continuum from high to low (Midgley and Dowling, 1978). Rogers (1983) stated that innovativeness might refer to how an individual is relatively onward in embracing new ideas than any other individual. Furthermore, Gozukara and Colakoglu (2016, p. 35) stated that “innovation is a particular mechanism through which entrepreneurs exploit environmental changes as an opportunity toward a new business.”

Risk-taking includes courageous activities, orientation for borrowings, or investing substantial resources to set forth into dithering environments (Rauch et al., 2009). Bin and Park (2002) stated that there is a difference between entrepreneurs with a high risk-taking inclination and those with an aversion to risk. The first ones tend to make decisions faster to capitalize on opportunities, while the second ones tend to make more prudent decisions. The literature underpins that younger individuals are more predisposed to risk-taking actions than older ones (Jianakoplos and Bernasek, 2006; Sharland, 2006).

Proactiveness involves creating change, not anticipating it, which implies the quest for the following features of behaviors: “change opportunities,” setting “change-oriented goals,” “anticipating and preventing problems,” “doing different things or doing things differently,” “taking action,” “persevering,” and “achieving results” (Bateman and Crant, 1999, pp. 65–66). Proactiveness is examined also as the “opposite of reactiveness” (Bell, 2019, p. 821), can imply a forward-looking perspective and can materialize things by necessary actions and means (Lumpkin and Dess, 1996).

### Individual entrepreneurial orientation and entrepreneurial intention

In terms of the inclusion of IEO as a construct of entrepreneurial intention and the interrelations between them, there are several researchers that investigated this issue.

In this line, Awang et al. (2016) set up that, for the students from Malaysia, the IEO positively influenced their entrepreneurial intention. The same positive relationship between IEO (by the three-dimensional construct, namely, innovativeness, risk-taking, and proactiveness) and entrepreneurial intention among business students at Indonesian universities was drawn to attention by So et al. (2017). Langkamp Bolton and Lane (2012, p. 216), which set the measurement of entrepreneurial orientation for university students by the three dimensions, namely, “innovativeness, risk-taking, and proactiveness,” dwelled on significant statistical correlations between each dimension of the IEO and entrepreneurial intention.

Therefore, to replicate and confirm early results linking the three dimensions of IEO with intentions and to bring new empirical evidence on the modelers and deterring factors of students’ entrepreneurial intention in the context of the COVID-19 pandemic, the following research hypotheses are configured and targeted:

H1: Risk-taking will have a direct, positive, and significant influence on entrepreneurial intention;

H2: Innovativeness will have a direct, positive, and significant influence on entrepreneurial intention;

H3: Proactiveness will have a direct, positive, and significant influence on entrepreneurial intention.

### Entrepreneurial university environment

The concept of “entrepreneurial environment” grasps a merger of factors/dimensions that may enhance entrepreneurship (Fogel, 2001; Coduras and Autio, 2013). An entrepreneurial university environment can be defined as the combination of factors pertaining to a university such as education programs, subjects, seminars, or courses that contribute to entrepreneurial thinking formation and the development of entrepreneurial competencies among its students. In a straightforward way, Hannon (2013, pp. 12–13) assigned “two key dimensions” to explain the entrepreneurial university concept, namely, (i) the foremost organization capable of offering solutions to governments, employers, and students and their parents in the multi-faceted implications and pressures that entrepreneurial conjuncture may create in a specific period, within its continuous adaptation “to better align with its environment” and as the main driver to inspire entrepreneurial reflection and (ii) the capacity to engender the ambiance through which the “development of entrepreneurial mindsets and behaviors is embedded, encouraged, supported, incentivized, and rewarded.”

Considering that the education provided by universities shapes the career selection of students, higher education institutions can be perceived as a potential source for future entrepreneurs. Nowadays, most universities invest significant amounts of money in designing a solid entrepreneurship education for their students. Hence, entrepreneurship education is a key factor in the entrepreneurial university environment as it supports the acquirement of skills, knowledge, and mindset/attitudes that develop the entrepreneurial intentions and entrepreneurial behavior of students.

The entrepreneurial university environment encompasses both public and private universities, whereas entrepreneurial education is delivered by a large number of professors, with Ph.D. degrees, academic stamina, keenly involved in both scientific research and teaching activities, but also by specialists in various fields and business representatives in a dual education system, that are best qualified to shape students’ entrepreneurial intention. The constant interaction between higher education institutions and companies is essential to make the students aware of the economic, social, and technical realities of the labor market and the business environment (Barral et al., 2018). The COVID-19 pandemic has reinforced this paradigm and brought new challenges to universities in providing the necessary skills, desires, self-efficacy, and viability for students that are essential to successfully engage in the world of online work or to be self-employed and become entrepreneurs. Therefore, the consolidation of the interaction between government/policymakers, higher education institutions, and business representatives (e.g., “Triple Helix” defined by Audy, 2006) is essential to enhance the entrepreneurial intentions and entrepreneurial spirit among students. A strategic partnership and a dual education system ensure practical entrepreneurial classes for students, a deeper understanding of the labor market and of the business environment, and significantly shape the innovativeness of students, a key coordinate of entrepreneurial intention.

In this perspective, according to Lüthje and Franke (2002) and Cera et al. (2020), a notable focus was placed on formal entrepreneurship education at the university level as individuals with higher formal entrepreneurship education present increased intentions to start a business. However, external factors like entrepreneurship education influence students’ awareness that underlie their entrepreneurial intentions and can influence students’ entrepreneurship awareness. Through the academic engagement of both educators and learners, in connection with business representatives, policy, and governance officials, entrepreneurship education can notably shape and enhance the entrepreneurial intentions and actions of youths in terms of business establishment. The specific means of intervention encompass the wide spectrum of entrepreneurship education that covers innovative teaching techniques, vocational training, simulations, skill development, and curriculum design.

Moreover, if higher education institutions strengthen student learning to acquire knowledge and skills related to entrepreneurship, it may ensure and enhance “entrepreneurial cognition and activities” for students (Hassan et al., 2021, p. 406). According to Ibrahim and Lucky (2014), the environment within the university can become an influential factor in the formation of the entrepreneurial ecosystem. Kobylińska and Lavios (2020, p. 119) entail the importance of the university entrepreneurship ecosystem in shaping entrepreneurial intention as it comprises “many entities related to education, research, and social networks that contribute to growing entrepreneurial activity.” In this context, the recognition of the university environment as entrepreneurial is essential for students.

Many studies have shown that entrepreneurship education could stimulate students to be proactive, risk-takers rather than risk-averse, supports the decision-making process of creating new venture, and therefore leads to the entrepreneurial intention of students, as Wu and Wu (2008); Dehghanpour Farashah (2013), and Zhang et al. (2014) also proved. Wu and Wu (2008) suggested that the diversity of educational backgrounds of students may induce differences in entrepreneurial intentions and that higher education institutions need to design flexible approaches to entrepreneurial education for different groups of students tailored to their educational background and matching their needs. A strong entrepreneurial academic education nurtures the entrepreneurial intentions of university students and contributes to the sustainable competitive advantage of countries. Similarly, Sun et al. (2017) found that the four components of entrepreneurial education, namely, “Why, What, How, and Who,” influenced the entrepreneurial intention of students from Hong Kong. In this context, Hannon (2013, p. 14) inferred that the paramount challenge for the universities to become entrepreneurial is set on “How” they make and “create effective environments for developing entrepreneurial capacities in their staff and students.” A specific way in which universities may act for the entrepreneurial environment is the foundation of national centers to orient and support the synergy between universities and entrepreneurs/labor market, such as “the National Centre for Entrepreneurship in Education, NCEE” in the United Kingdom (Hannon, 2013).

The survey results conducted by Tung (2011) revealed that educational and structural support factors influence students’ entrepreneurial intention. Undoubtedly, entrepreneurship education and the university environment encourage entrepreneurial behavior and mindset among students.

Thus, the following hypothesis is defined and targeted:

H4: Entrepreneurial university environment will have a direct, positive, and significant influence on entrepreneurial intention.

As regards the relation between entrepreneurial personality (i.e., innovativeness) and entrepreneurial intention, Roy et al. (2017) and Hassan et al. (2021) could not prove that there is a direct path from one to the other, but when perceived self-efficacy (i.e., entrepreneurial motivation) was interposed between them, as a mediator factor, the effect was favorable.

Accordingly, along with the direct influence of innovativeness on increasing entrepreneurial intention, it has also been proposed that an entrepreneurial university environment will mediate the relationships between innovativeness and entrepreneurial intention. Therefore, the following hypothesis emerges:

H5: The positive relationship between innovativeness and entrepreneurial intention is mediated by the entrepreneurial university environment.

Besides these five hypotheses built on the research objective of this paper, for assessing the entrepreneurial university environment there are also eight mainstays/pillars proposed at the European level, by the European Commission in partnership with the OECD, which created “a free self-assessment tool for all types of higher education institution, HEInnovate” (HEInnovate, 2022).^1^ The eight pillars designed for the evaluation of the entrepreneurial university environment are as follows: “Leadership and governance”; “Organizational Capacity: Funding, People and Incentives”; “Entrepreneurship Teaching and Learning”; “Preparing and Supporting Entrepreneurs”; “Digital Transformation and Capability”; “Knowledge Exchange and Collaboration”; “The International Institution”; and “Measuring the Impact” (HEInnovate, 2022).^2^ HEInnovate acts like a “guiding framework for the entrepreneurial university” (Hannon, 2013, p. 14) that helps them “to review what they do and how and the effects on the enhancement or inhibition of the development of entrepreneurial capacities that will underpin innovation capacity.” In Romania, according to HEInnovate (2022, see text footnote 2) metrics, in July 2022, 150 higher education institutions were registered through the platform, totaling 8,399 self-assessments. Therefore, this online guideline may provide a useful tool for enhancing the entrepreneurial university environment.

## Data and methodology

### Data collection procedure and descriptive analysis

The data processed in the empirical analysis were compiled using an online questionnaire (Supplementary Table 1) applied to a sample of 1,411 students at the West University of Timi?oara, one of Romania’s most prominent universities. First, to contact the responding students, a list of e-mails from 16,606 students was used. Afterward, an invitation to complete an online questionnaire was sent by e-mail to the students with a cover letter that explained the purpose of the survey, the completion time, and the confidentiality policy. In addition, to improve the response rate, two reminder e-mails were sent. The data were collected for 4 months, from November 2020 to February 2021. Out of the 1,426 responses received, 1,411 usable questionnaires with complete information were retained for the empirical analysis. Thereby, the final response rate was 8.5%. The sample size was relevant in providing robust results and policy guidelines, being consistent with other studies performed on the same topic (e.g., Turker and Sonmez Selcuk, 2009; Langkamp Bolton and Lane, 2012; Roy et al., 2017; Duong et al., 2021).

The respondents had to answer with “yes” or “no” to the question: “Do you consider that the economic situation in Romania due to the COVID-19 pandemic inhibits the manifestation of the entrepreneurial spirit?” Regarding this question, approximately three-quarters of the students (71.8%) in the sample answered with “yes.” In contrast, only 28.2% of the respondents had considered that the COVID-19 pandemic does not inhibit the manifestation of the entrepreneurial spirit. The students’ age ranged from 18 years to 57 years with an average of 22.42 years. Approximately two-thirds of the students were female (68%) and were living in urban areas (66.8%). Finally, most of the respondents were bachelor level students (82.6%), while the rests were master level or Ph.D. students (Table 1).

**TABLE 1:** Sample characteristics.

Features	Share of total sample (%)
***Age***	
18–21 years	70.4%
22–25 years	14.4%
26–35 years	8.8%
Over 36 years	6.4%
***Gender***	
Male	32%
Female	68%
***Area of living***	
Urban	66.8%
Rural	33.2%
***Studies***	
Bachelor	82.6%
Master	15.2%
Ph.D.	2.2%

Authors’ process in SPSS.

### Measurements

The research used multi-item scales, adapted from the literature underpinnings (Linan and Chen, 2009; Langkamp Bolton and Lane, 2012; Langkamp Bolton, 2012; So et al., 2017; Akhmetshin et al., 2019), to assess the five constructs included in the research model (Supplementary Table 1).

First, to assess the three dimensions of the IEO, namely, *risk-taking* (three items), *innovativeness* (four items), and *proactiveness* (three items), the current study used the scale developed by Langkamp Bolton and Lane (2012) and Langkamp Bolton (2012). The scales used to measure *risk-taking* (Cronbach’s alpha = 0.815), *innovativeness* (Cronbach’s alpha = 0.830), *and proactiveness* (Cronbach’s alpha = 0.741) were reliable.

Next, the *Entrepreneurial university environment* construct was captured using Akhmetshin et al.’s (2019) five-item scale. This scale was also reliable (Cronbach’s alpha = 0.847). Finally, the dependent variable, the *entrepreneurial intention*, was measured using nine items adapted from the studies led by Linan and Chen (2009) and So et al. (2017). The reliability of the *entrepreneurial intention* construct was found to have a Cronbach’s alpha = 0.951. Responses were measured on a five-point Likert scale, anchored at 1 (total disagreement) to 5 (total agreement).

### Methodology

The methodological endeavor is configured through the lens of SEM, as a modern and complex technique, on the one hand, and graphical Markov model, namely, a GGM, on the other hand. For the entire methodological endeavor performed in this paper, three econometric packages were used, namely, SPSS, Stata, and RStudio.

**FIGURE 1:**
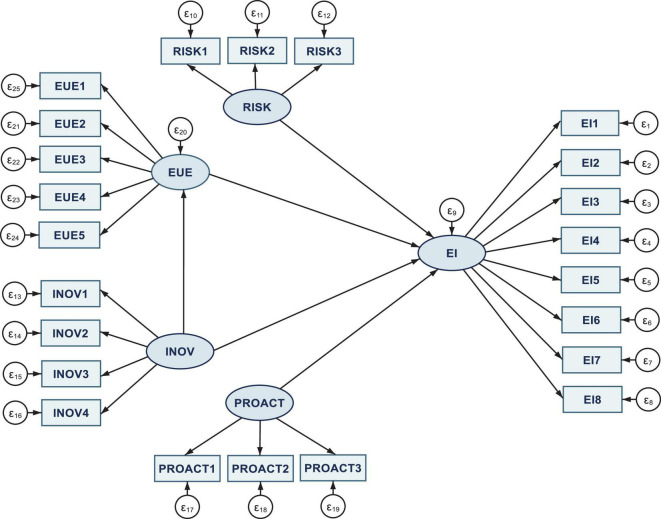
General configuration of the SEM model. Authors’ contribution in Stata 16.

The general design of the *SEM model*, performed in Stata, is detailed in Figure 1. It captures, in a gradual framework, the direct, indirect, and total interlinkages, to process cross-sectional data compiled through the MLE method, between three major dimensions of entrepreneurial intention (EI), namely, risk-taking (RISK), proactiveness (PROACT), and innovativeness (INOV), along with the mediated impact of the entrepreneurial university environment (EUE), as well as their further impact on entrepreneurial intention (EI).

In this framework, both measurement and structural components fall under the framework of the SEM model design, SEM being applied by other researchers as well in approaching similar topics (e.g., Duong et al., 2021). SEM allowed associating multiple measures for each of the three dimensions that influence the entrepreneurial intention, along with the decisive role of the entrepreneurial university environment (EUE) and the EI itself, with a latent construct.

Further, there is an assessment of all these interconnections and explicitly model measurement errors to derive unbiased estimates for the relations between latent constructs. Hence, the five research hypotheses employed in this study are tested using the SEM technique to examine the direct, indirect, and total relationships between the variables (both measured and latent), consistent with the theoretical model and assumptions. Furthermore, the mediation or indirect effect is essential for the research design in a three-fold setting between *INOV-EUE* and *EI*, being captured through SEM as another advantage and technical advancement of this multivariate modeling technique. Gunzler et al. (2013, p. 392) showed that “the indirect effect describes the pathway from the exogenous variable to the outcome through the mediator. The total effect is the sum of the direct and indirect effects of the exogenous variable on the outcome.”

The general SEM model is being processed on the entire sample (SEM 1) and on two sub-samples, configured relying on the fact that the respondents acknowledged (SEM 2) or not the impact of the COVID-19 pandemic on the entrepreneurial intention (SEM 3). Hence, there are three sets of SEM models and associated results.

In the second part of the empirical analysis, SEM is complemented by *network analysis through* GGMs, to further ensure the robustness of the results and to capture the direct positive connections between the measured variables and the intensity of these linkages, while controlling for the other variables in the dataset. The GGMs, performed in RStudio package, are estimated through the extended Bayesian information criteria (EBIC) with graphical least absolute shrinkage and selection operator (glasso) and through partial correlation (PCOR).

## Results and discussion

The SEM models designed in the current research have two fundamental parts: (i) a measurement representation, capturing the connections between measured and latent variables, and (ii) and a structural wedge, grasping the interdependence between factors (e.g., the latent variables).

**FIGURE 2:**
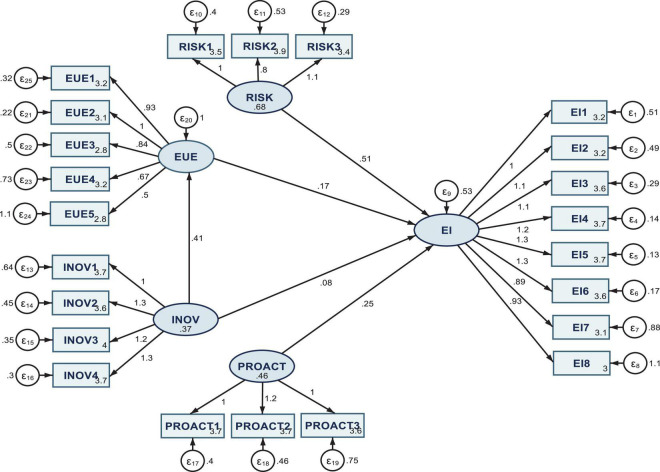
Results of the SEM 1, processed on the entire sample. Authors’ research in Stata 16.

**FIGURE 3:**
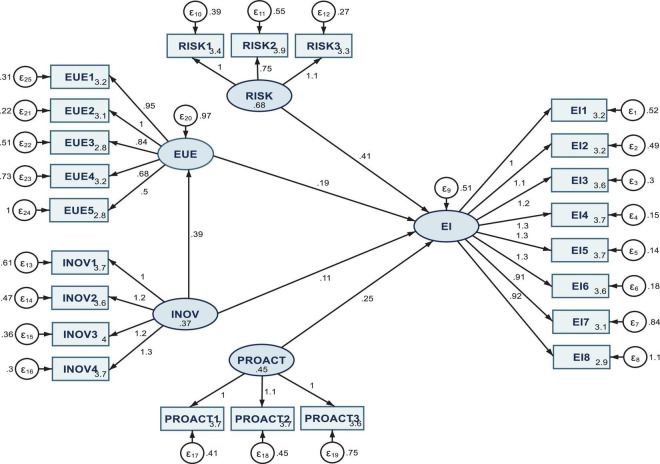
Results of SEM 2 model processed on the sub-sample acknowledging COVID-19 modelers and influence on the entrepreneurial intention. Authors’ research in Stata 16.

**FIGURE 4:**
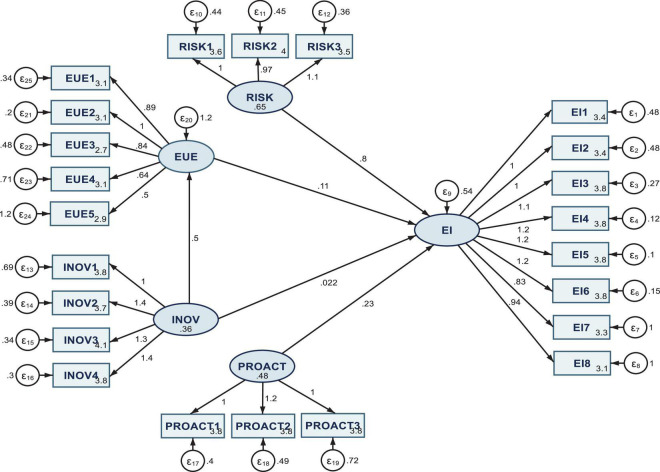
Results of SEM 3 model processed on the sub-sample considering that COVID-19 does not influence the entrepreneurial intention. Authors’ research in Stata 16.

The measurement model results are entailed in Figure 2, for the entire sample (SEM 1), and Figure 3 (SEM 2) and Figure 4 (SEM 3), respectively, for the two sub-samples acknowledging or not COVID-19 influence on the entrepreneurial intention.

The main results of the structural parts are detailed in Table 2 and Supplementary Table 2. The Supplementary material also details the results of several robustness check tests performed for the SEM models: “Cronbach’s alpha” for all SEM models (Supplementary Table 3), with a total alpha scale around 0.93 for all models; “Wald’s tests for equations associated with the SEM models” (Supplementary Table 4), with a *p*-value of 0.000 (registered value) for all equations; and “the goodness-of-fit tests” (Supplementary Table 5), namely, “likelihood ratio (model vs. saturated, baseline vs. saturated), information criteria (Akaike’s and Bayesian), baseline comparison (comparative fit index, CFI, and Tucker–Lewis index, TLI), size of residuals (standardized root mean squared residual, SRMR, and the coefficient of determination, CD, which is over 0.99 for all models).” Therefore, the outcomes of these tests highlight that all SEM results are robust and accurate, being suitable for a proper economic interpretation.

**TABLE 2:** Indirect and total effects captured in the SEM models by MLE method.

	SEM 1 (entire sample)	SEM 2 (with COVID-19 influence)	SEM 3 (without COVID-19 influence)
**Indirect effects (structural)**			
EI ->	β/se	β/se	β/se
EUE	0 (no path)	0 (no path)	0 (no path)
RISK	0 (no path)	0 (no path)	0 (no path)
INOV	0.0685*** (0.0125)	0.0729*** (0.0151)	0.0545*** (0.0231)
PROACT	0 (no path)	0 (no path)	0 (no path)
**Total effects (structural)**			
EI ->	β/se	β/se	β/se
EUE	0.165*** (0.0220)	0.188*** (0.0261)	0.109** (0.0397)
RISK	0.508*** (0.0412)	0.412*** (0.0455)	0.804*** (0.0987)
INOV	0.0801 (0.0531)	0.115 (0.0611)	0.0222 (0.105)
PROACT	0.254*** (0.0458)	0.246*** (0.0522)	0.230* (0.0910)

“Standard errors in parentheses, *p < 0.05, **p < 0.01, ***p < 0.001.” Authors’ contribution in Stata 16.

According to the five hypotheses framed on the literature underpinnings, the first four hypotheses assess the implications of *risk-taking* (RISK), innovativeness (INOV), proactiveness (PROACT), and entrepreneurial university environment (EUE) on the entrepreneurial intention (EI). For the fifth research hypothesis, the current study considers the mediation effect of the entrepreneurial university environment (EUE) in enhancing the positive and significant innovativeness (INOV) impact on entrepreneurial intention (EI). In other words, the research entails whether the impact of the explanatory variable (innovativeness) on the outcome (entrepreneurial intention) can be mediated by a change in the mediating variable (entrepreneurial university environment). Hence, the indirect and total effects are also captured in this framework.

After processing the SEM model through the MLE method, the first set of results (SEM 1, Table 2 and Figure 2) grasp positive coefficients and favorable connections between all considered variables. Hence, risk-taking (RISK) has a direct, positive, and significant impact on EI (*H1 is fulfilled*), proactiveness (PROACT) has a direct, positive, and significant influence on *EI* (*H3 is fulfilled*), and entrepreneurial university environment (EUE) has a direct, positive, and significant influence on *EI* (*H4 is fulfilled*). These results are in line with those revealed by Langkamp Bolton and Lane (2012); Awang et al. (2016), and So et al. (2017), which stated that each of the three dimensions of IEO, namely, risk-taking, innovativeness, and proactiveness, significantly shaped students’ entrepreneurial intention.

The same positive estimated coefficient regarding the direct impact of innovativeness (INOV) on EI can be noted. However, in this case, the impact is not significant from a statistical point of view. Along these lines, there is an acknowledgment of *H5* as regards the mediation (indirect) effect of the entrepreneurial university environment (EUE) in shaping the impact of INOV on EI.

When capturing these mediation effects, the estimated coefficients associated with INOV remain positive and become highly statistically significant, as shown in Table 2. Therefore, *H2* is partially fulfilled and *H5* is being fulfilled.

These results are in line with those of previous studies. The one performed by Roy et al. (2017) showed no direct link between innovativeness and entrepreneurial intention, but instead, EI is fully mediated by other credentials (such as perceived self-efficacy). In this regard, the results evidence the decisive positive role of the entrepreneurial university environment in shaping students’ EI, as also highlighted by relevant literature (e.g., Lüthje and Franke, 2002; Tung, 2011; Zhang et al., 2014; Sun et al., 2017) grasping that tertiary education, namely, the university entrepreneurial education and training, underlies students’ entrepreneurial intentions, as it fosters the attainment of advanced knowledge and abilities associated with the start-up process, as prerequisites for successful entrepreneurship engagement of students [as also entailed by Kobylińska and Lavios (2020) and Hassan et al. (2021)]. A notable focus in this regard was placed by Cera et al. (2020) on formal entrepreneurship education at the university level, as individuals with higher formal entrepreneurship education present increased intentions to start a business.

The COVID-19 pandemic does not seem to bring notable pitfalls to the entrepreneurial intention among the students considered in this research. The respondents acknowledge the severe impact that COVID-19 has on the business environment. However, the results (Figure 3 and Table 2, model SEM 2) entail that, even in this particular case, all four dimensions of risk-taking, proactiveness, innovativeness, and entrepreneurial university environment positively shape the entrepreneurial intention (with limitation on INOV, where its direct and total effect on EI, captured through the estimated coefficients, is positive, but the coefficients are not significant from a statistical point of view). Even in this scenario, when the mediation effect of EUE is considered for the impact of INOV on EI, the results are statistically significant at the 0.1% threshold, and the estimated coefficient is positive. These results are in line with those obtained by Ratten (2021) that underlined as a possible solution to reduce unemployment by encouraging young people to indulge in entrepreneurial activities, in synergy with the acknowledgment of innovation in order to stand up to the difficulties caused by the COVID-19 pandemic.

When the sample of a student considering that COVID-19 does not influence the entrepreneurial intention is processed (Figure 4 and Table 2, model SEM 3), the results highlight the same positive and significant estimated coefficients associated with the three dimensions modeling the entrepreneurial intention.

Nevertheless, the risk-taking dimension has a stronger shaping impact on the entrepreneurial intention than the previous two SEM/samples (total and without the COVID-19 influence). Furthermore, the innovativeness tends to deter slightly. Still, the EUE strongly mediates its influence on EI, even though it is slightly less significant (at the 1% threshold) than the previous two SEM models.

**FIGURE 5:**
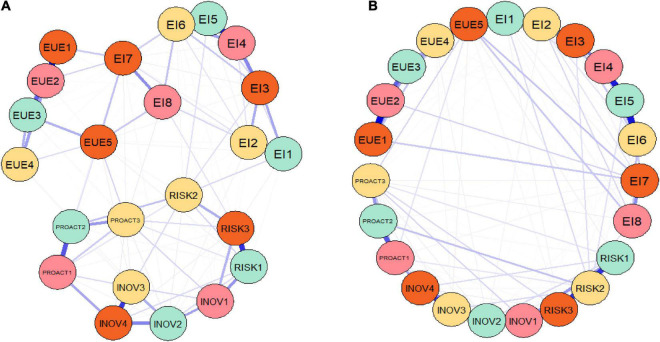
Results of the GGMs processed on the entire sample. **(A)** EBICglasso method. **(B)** PCOR method. Authors’ research in RStudio.

In addition to the SEM models, the current research also configured a network analysis performed through a graphical model, namely, a GGM, estimated through the EBIC with graphical least absolute shrinkage and selection operator (EBICglasso), as presented in Figure 5A, and through PCOR, as entailed in Figure 5B.

The variables are captured as nodes in this graphical setting (GGM network), the blue edges entail positive partial correlations, and the red ones reveal negative connections, while the absolute strength of a partial correlation is represented by the width and saturation of a line (Epskamp et al., 2018).

Acknowledging the results of the GGM (Figure 5), it can be noted that there are positive correlations among all considered variables (only blue lines among variables), and the items defining the entrepreneurial intention (EI) are strongly connected with *EUE* item, but also with risk-taking (RISK) and proactiveness (PROACT) items, and less with innovativeness (INOV) credentials.

Therefore, GGMs complement and validate previous SEM results and enhance the essential role that the entrepreneurial university environment plays in shaping the entrepreneurial intention of youths/students through significantly enhancing their innovativeness, particularly during the challenging COVID-19 pandemic, but also post-pandemic times.

## Conclusion

This study was conducted to assess the main modelers, drivers, and deterring factors of entrepreneurial intention for Romanian university students during the COVID-19 pandemic. The framework designed in this study centered on the antecedents of entrepreneurial intention among students, embedding three focal constructs, namely, risk-taking, proactiveness, and innovativeness, as measurement instruments attested by the literature (Langkamp Bolton and Lane, 2012), with a keen focus on the mediation effect of the entrepreneurial university environment.

Through a combination of modern and advanced econometric techniques relying on the structural equation (SEM) and network analysis with GGMs, the current research, through the five hypotheses tested, brings forward that the entrepreneurial intention of students from the west part of Romania was significantly and positively shaped by risk-taking, proactiveness, and entrepreneurial university environment in this difficult pandemic outbreak, while innovativeness induced a positive and significant influence on entrepreneurial intention only through the mediated impact of the entrepreneurial university environment.

These results are encouraging and shed new light for policymakers, as well as for teachers, on the decisive role played by tertiary entrepreneurship education in shaping youths’ entrepreneurial intentions. Therefore, by including and extending the entrepreneurship curriculum by the Romanian universities, irrespective of specialization fields, students can enhance their capabilities for risk-taking behavior, increasing their awareness of innovativeness by interacting with successful entrepreneurs from the labor market, motivations, and proactiveness for entrepreneurial activities, “by stimulating the development of an entrepreneurial mindset,” as Hassan et al. (2021, p. 413) highlighted for Indian universities. Based on these findings, as a recommendation for policymakers from Romania, an agreed standard structure at the national level of entrepreneurship curriculum would provide structural support for cultivating necessary entrepreneurship knowledge and skills. Withal, universities can perform new teaching methods by involving mentoring programs in the field of entrepreneurship. Moreover, configuring a national center for entrepreneurial education would ensure permanent contact and connection between universities and business representatives and therefore would contribute to the development of entrepreneurial intentions among young people. Also, to keep in line with European and global trends in entrepreneurship education, universities are encouraged to permanently make self-assessments on the HEInnovate platform.

The main research limitation embeds the fact that the sample was configured only with students from western Romania, hence partially restraining a generalization of the results. However, this study was intended to strengthen the knowledge in this scientific field with new empirical evidence and theoretical groundings highlighting that risk-taking, proactiveness, and innovativeness mediated by entrepreneurial university education continue to be essential in framing students’ entrepreneurial intentions even during these challenging COVID-19 pandemic times. Future research aims to expand the sample at the national level in Romania and consider students from various universities in other European Union member states, in a comparative approach. Related to HEInnovate (2022) platform, another future research direction is set on assessing the entrepreneurial university environment by the means of all eight pillars proposed, associated with “leadership, staffing, and links with business.”

## Data availability statement

The original contributions presented in this study are included in the article/Supplementary material, further inquiries can be directed to the corresponding authors.

## Author contributions

SMT, GGN, MC, CE, and OD: conceptualization. GGN, SMT, and MC: methodology, validation, and formal analysis. GGN and SMT: software. SMT and CE: data collection. MC, GGN, and OD: investigation. SMT, GGN, MC, and OD: writing—original draft preparation, review, and editing. All authors contributed to the article and approved the submitted version.

## Footnotes

1
https://www.heinnovate.eu/en/about/heinnovate

2
https://www.heinnovate.eu/en
