# Supplementary material for: Modelers of students’ entrepreneurial intention during the COVID-19 pandemic and post-pandemic times: The role of entrepreneurial university environment
Source: Front Psychol. 2022 Sep 6;13:976675. doi: 10.3389/fpsyg.2022.976675 (PMC9485888; doi:10.3389/fpsyg.2022.976675)
Supplement: Supplementary file 1 [file Table_1.DOCX]

Supplementary Material

**Table S1.** Questionnaire items and their source of adoption

|  | Constructs and measuring items | Sources |
| --- | --- | --- |
| Please indicate your level of agreement with the following sentences (total disagreement: 1, total agreement: 5) | | |
| IEO | “Individual Entrepreneurial Orientation (IEO)”  *Dimensions* | Langkamp Bolton (2012, p. 94) |
|  |  |  |
| RISK | *“Risk-taking”* |  |
| RISK1 | “I like to take bold action by venturing into the unknown.” |  |
| RISK2 | “I am willing to invest a lot of time and/or money on something that might yield a high return.” |  |
| RISK3 | “I tend to act “boldly” in situations where risk is involved.” |  |
|  |  |  |
| INOV | *“Innovativeness”* |  |
| INOV1 | “I often like to try new and unusual activities that are not typical but  not necessarily risky.” |  |
| INOV2 | “In general, I prefer a strong emphasis in projects on unique, one-of-a-kind approaches, rather than revisiting tried and true approaches used before.” |  |
| INOV3 | “I prefer to try my own unique way when learning new things rather than doing it like everyone else does.” |  |
| INOV4 | “I favor experimentation and original approaches to problem solving rather than using methods others generally use for solving their problems.” |  |
|  |  |  |
| PROACT | *“Proactiveness”* |  |
| PROACT1 | “I usually act in anticipation of future problems, needs or changes.” |  |
| PROACT2 | “I tend to plan ahead on projects.” |  |
| PROACT3 | “I prefer to ‘step-up’ and get things going on projects rather than sit and wait for someone else to do it.” |  |
|  |  |  |
| EUE | *“Entrepreneurial university environment”* | Akhmetshin et al. (2019, p. 5) |
| EUE1 | “I can characterize the environment of my university as formative of entrepreneurial thinking.” |  |
| EUE2 | “My current education program meets my needs for the formation of entrepreneurial thinking.” |  |
| EUE3 | “I consider the introduction of the new subjects to be necessary for the formation of entrepreneurial thinking.” |  |
| EUE4 | “I attend additional training/courses/seminars for the formation of entrepreneurial thinking.” |  |
| EUE5 | “I consider my current level of entrepreneurial thinking to be sufficient for a successful start in a profession” |  |
|  |  |  |
| EI | *“Entrepreneurial intention”* | So et al. (2017, p. 283) |
| EI1 | “I am ready to do anything to be an entrepreneur.” |  |
| EI2 | “My professional goal is to be an entrepreneur.” |  |
| EI3 | “I will make every effort to start and run my own business.” |  |
| EI4 | “I am determined to create a business venture in the future.” |  |
| EI5 | “I do not have doubts about ever starting my own business in the future.” |  |
| EI6 | “I have very seriously thought of starting a business in the future.” |  |
| EI7 | “I have a strong intention to start a business in the future.” |  |
| EI8 | “My qualification has contributed positively towards my interest in starting a business.” |  |
| EI9 | “I had a strong intention to start my own business before I started with my qualification.” |  |

Source: Authors’ compilation based on Langkamp Bolton (2012), Akhmetshin et al*.* (2019) and So et al*.* (2017)

**Table S2.** Detailed results of all SEM models, MLE – maximum likelihood estimator

|  | (1) | (2) | (3) |
| --- | --- | --- | --- |
|  | SEM 1 – overall | SEM 2 – with COVID-19  modelers of EI | SEM 3 – without COVID-19 modelers of EI |
| EI |  |  |  |
| EUE | 0.165^***^ (0.0220) | 0.188^***^ (0.0261) | 0.109^**^ (0.0397) |
| RISK | 0.508^***^ (0.0412) | 0.412^***^ (0.0455) | 0.804^***^ (0.0987) |
| INOV | 0.0801 (0.0531) | 0.115 (0.0611) | 0.0222 (0.105) |
| PROACT | 0.254^***^ (0.0458) | 0.246^***^ (0.0522) | 0.230^*^ (0.0910) |
| EUE |  |  |  |
| INOV | 0.415^***^ (0.0529) | 0.388^***^ (0.0608) | 0.502^***^ (0.109) |
| EI1 |  |  |  |
| EI | 1 (.) | 1 (.) | 1 (.) |
| _cons | 3.244^***^ (0.0302) | 3.194^***^ (0.0347) | 3.369^***^ (0.0608) |
| EI2 |  |  |  |
| EI | 1.066^***^ (0.0290) | 1.077^***^ (0.0366) | 1.041^***^ (0.0475) |
| _cons | 3.235^***^ (0.0312) | 3.181^***^ (0.0359) | 3.374^***^ (0.0626) |
| EI3 |  |  |  |
| EI | 1.150^***^ (0.0277) | 1.188^***^ (0.0357) | 1.074^***^ (0.0432) |
| _cons | 3.636^***^ (0.0306) | 3.586^***^ (0.0357) | 3.761^***^ (0.0597) |
| EI4 |  |  |  |
| EI | 1.238^***^ (0.0273) | 1.278^***^ (0.0353) | 1.162^***^ (0.0419) |
| _cons | 3.714^***^ (0.0307) | 3.674^***^ (0.0357) | 3.817^***^ (0.0606) |
| EI5 |  |  |  |
| EI | 1.291^***^ (0.0283) | 1.334^***^ (0.0367) | 1.209^***^ (0.0432) |
| _cons | 3.702^***^ (0.0318) | 3.654^***^ (0.0370) | 3.824^***^ (0.0626) |
| EI6 |  |  |  |
| EI | 1.295^***^ (0.0289) | 1.339^***^ (0.0374) | 1.209^***^ (0.0442) |
| _cons | 3.613^***^ (0.0323) | 3.556^***^ (0.0376) | 3.759^***^ (0.0634) |
| EI7 |  |  |  |
| EI | 0.888^***^ (0.0320) | 0.914^***^ (0.0396) | 0.834^***^ (0.0550) |
| _cons | 3.135^***^ (0.0325) | 3.089^***^ (0.0375) | 3.254^***^ (0.0651) |
| EI8 |  |  |  |
| EI | 0.928^***^ (0.0351) | 0.918^***^ (0.0440) | 0.940^***^ (0.0576) |
| _cons | 2.972^***^ (0.0355) | 2.914^***^ (0.0412) | 3.118^***^ (0.0694) |
| RISK1 |  |  |  |
| RISK | 1 (.) | 1 (.) | 1 (.) |
| _cons | 3.471^***^ (0.0277) | 3.412^***^ (0.0325) | 3.621^***^ (0.0523) |
| RISK2 |  |  |  |
| RISK | 0.803^***^ (0.0334) | 0.754^***^ (0.0384) | 0.973^***^ (0.0749) |
| _cons | 3.883^***^ (0.0263) | 3.856^***^ (0.0304) | 3.952^***^ (0.0517) |
| RISK3 |  |  |  |
| RISK | 1.110^***^ (0.0362) | 1.099^***^ (0.0440) | 1.133^***^ (0.0685) |
| _cons | 3.372^***^ (0.0283) | 3.311^***^ (0.0328) | 3.528^***^ (0.0545) |
| INOV1 |  |  |  |
| INOV | 1 (.) | 1 (.) | 1 (.) |
| _cons | 3.718^***^ (0.0268) | 3.668^***^ (0.0312) | 3.844^***^ (0.0513) |
| INOV2 |  |  |  |
| INOV | 1.276^***^ (0.0582) | 1.230^***^ (0.0670) | 1.400^***^ (0.120) |
| _cons | 3.598^***^ (0.0273) | 3.555^***^ (0.0320) | 3.709^***^ (0.0522) |
| INOV3 |  |  |  |
| INOV | 1.217^***^ (0.0571) | 1.179^***^ (0.0657) | 1.324^***^ (0.118) |
| _cons | 4.001^***^ (0.0253) | 3.964^***^ (0.0295) | 4.093^***^ (0.0491) |
| INOV4 |  |  |  |
| INOV | 1.318^***^ (0.0610) | 1.286^***^ (0.0707) | 1.418^***^ (0.124) |
| _cons | 3.744^***^ (0.0259) | 3.719^***^ (0.0302) | 3.809^***^ (0.0505) |
| PROACT1 |  |  |  |
| PROACT | 1 (.) | 1 (.) | 1 (.) |
| _cons | 3.736^***^ (0.0248) | 3.713^***^ (0.0291) | 3.794^***^ (0.0471) |
| PROACT2 |  |  |  |
| PROACT | 1.151^***^ (0.0609) | 1.149^***^ (0.0732) | 1.158^***^ (0.111) |
| _cons | 3.735^***^ (0.0275) | 3.719^***^ (0.0321) | 3.776^***^ (0.0535) |
| PROACT3 |  |  |  |
| PROACT | 1.018^***^ (0.0551) | 1.007^***^ (0.0655) | 1.029^***^ (0.101) |
| _cons | 3.614^***^ (0.0295) | 3.552^***^ (0.0346) | 3.774^***^ (0.0556) |
| EUE2 |  |  |  |
| EUE | 1 (.) | 1 (.) | 1 (.) |
| _cons | 3.128^***^ (0.0304) | 3.125^***^ (0.0352) | 3.136^***^ (0.0602) |
| EUE3 |  |  |  |
| EUE | 0.843^***^ (0.0229) | 0.844^***^ (0.0280) | 0.838^***^ (0.0397) |
| _cons | 2.774^***^ (0.0301) | 2.787^***^ (0.0350) | 2.741^***^ (0.0584) |
| EUE4 |  |  |  |
| EUE | 0.670^***^ (0.0250) | 0.683^***^ (0.0306) | 0.642^***^ (0.0428) |
| _cons | 3.189^***^ (0.0294) | 3.205^***^ (0.0346) | 3.146^***^ (0.0555) |
| EUE5 |  |  |  |
| EUE | 0.497^***^ (0.0289) | 0.497^***^ (0.0348) | 0.498^***^ (0.0518) |
| _cons | 2.816^***^ (0.0309) | 2.776^***^ (0.0358) | 2.920^***^ (0.0606) |
| EUE1 |  |  |  |
| EUE | 0.931^***^ (0.0209) | 0.953^***^ (0.0255) | 0.885^***^ (0.0361) |
| _cons | 3.181^***^ (0.0299) | 3.203^***^ (0.0350) | 3.126^***^ (0.0574) |
| / |  |  |  |
| var(e.EI1) | 0.509^***^ (0.0203) | 0.520^***^ (0.0244) | 0.476^***^ (0.0358) |
| var(e.EI2) | 0.492^***^ (0.0198) | 0.495^***^ (0.0235) | 0.483^***^ (0.0365) |
| var(e.EI3) | 0.293^***^ (0.0125) | 0.302^***^ (0.0152) | 0.272^***^ (0.0215) |
| var(e.EI4) | 0.139^***^ (0.00707) | 0.147^***^ (0.00879) | 0.119^***^ (0.0114) |
| var(e.EI5) | 0.132^***^ (0.00714) | 0.141^***^ (0.00897) | 0.105^***^ (0.0111) |
| var(e.EI6) | 0.172^***^ (0.00845) | 0.180^***^ (0.0105) | 0.150^***^ (0.0139) |
| var(e.EI7) | 0.882^***^ (0.0339) | 0.838^***^ (0.0381) | 0.996^***^ (0.0718) |
| var(e.EI8) | 1.106^***^ (0.0424) | 1.131^***^ (0.0512) | 1.035^***^ (0.0750) |
| var(e.RISK1) | 0.398^***^ (0.0222) | 0.386^***^ (0.0268) | 0.444^***^ (0.0445) |
| var(e.RISK2) | 0.533^***^ (0.0247) | 0.549^***^ (0.0289) | 0.454^***^ (0.0494) |
| var(e.RISK3) | 0.285^***^ (0.0238) | 0.269^***^ (0.0285) | 0.355^***^ (0.0488) |
| var(e.INOV1) | 0.638^***^ (0.0272) | 0.614^***^ (0.0314) | 0.691^***^ (0.0537) |
| var(e.INOV2) | 0.448^***^ (0.0233) | 0.469^***^ (0.0282) | 0.390^***^ (0.0402) |
| var(e.INOV3) | 0.355^***^ (0.0188) | 0.361^***^ (0.0225) | 0.336^***^ (0.0340) |
| var(e.INOV4) | 0.302^***^ (0.0191) | 0.303^***^ (0.0227) | 0.299^***^ (0.0349) |
| var(e.PROACT1) | 0.404^***^ (0.0261) | 0.405^***^ (0.0309) | 0.401^***^ (0.0489) |
| var(e.PROACT2) | 0.459^***^ (0.0331) | 0.446^***^ (0.0388) | 0.493^***^ (0.0640) |
| var(e.PROACT3) | 0.747^***^ (0.0361) | 0.752^***^ (0.0425) | 0.720^***^ (0.0669) |
| var(e.EUE2) | 0.217^***^ (0.0165) | 0.222^***^ (0.0192) | 0.199^***^ (0.0318) |
| var(e.EUE3) | 0.501^***^ (0.0225) | 0.509^***^ (0.0268) | 0.483^***^ (0.0415) |
| var(e.EUE4) | 0.728^***^ (0.0294) | 0.733^***^ (0.0350) | 0.712^***^ (0.0538) |
| var(e.EUE5) | 1.081^***^ (0.0418) | 1.046^***^ (0.0478) | 1.152^***^ (0.0836) |
| var(e.EUE1) | 0.318^***^ (0.0174) | 0.309^***^ (0.0204) | 0.336^***^ (0.0330) |
| var(e.EI) | 0.534^***^ (0.0312) | 0.509^***^ (0.0357) | 0.537^***^ (0.0615) |
| var(e.EUE) | 1.026^***^ (0.0482) | 0.974^***^ (0.0548) | 1.155^***^ (0.0989) |
| var(RISK) | 0.684^***^ (0.0413) | 0.682^***^ (0.0488) | 0.646^***^ (0.0773) |
| var(INOV) | 0.372^***^ (0.0322) | 0.374^***^ (0.0377) | 0.355^***^ (0.0599) |
| var(PROACT) | 0.462^***^ (0.0358) | 0.453^***^ (0.0420) | 0.482^***^ (0.0685) |
| *N* | 1411 | 1013 | 398 |

Note: Standard errors in parentheses, ^*^ *p* < 0.05, ^**^ *p* < 0.01, ^***^ *p* < 0.001

Source: Authors’ contribution in Stata 16

**Table S3.** Cronbach’s alpha for all SEM models

|  | SEM 1 | | SEM 2 | | SEM 3 | |
| --- | --- | --- | --- | --- | --- | --- |
| Item | observation | Alpha | observation | Alpha | observation | Alpha |
| EI1 | 1411 | 0.9277 | 1013 | 0.9242 | 398 | 0.9345 |
| EI2 | 1411 | 0.9275 | 1013 | 0.9240 | 398 | 0.9342 |
| EI3 | 1411 | 0.9270 | 1013 | 0.9232 | 398 | 0.9343 |
| EI4 | 1411 | 0.9272 | 1013 | 0.9236 | 398 | 0.9341 |
| EI5 | 1411 | 0.9272 | 1013 | 0.9236 | 398 | 0.9341 |
| EI6 | 1411 | 0.9270 | 1013 | 0.9233 | 398 | 0.9341 |
| EI7 | 1411 | 0.9281 | 1013 | 0.9246 | 398 | 0.9350 |
| EI8 | 1411 | 0.9293 | 1013 | 0.9262 | 398 | 0.9355 |
| RISK1 | 1411 | 0.9297 | 1013 | 0.9264 | 398 | 0.9361 |
| RISK2 | 1411 | 0.9286 | 1013 | 0.9252 | 398 | 0.9350 |
| RISK3 | 1411 | 0.9291 | 1013 | 0.9256 | 398 | 0.9358 |
| INOV1 | 1411 | 0.9305 | 1013 | 0.9271 | 398 | 0.9372 |
| INOV2 | 1411 | 0.9299 | 1013 | 0.9263 | 398 | 0.9369 |
| INOV3 | 1411 | 0.9310 | 1013 | 0.9277 | 398 | 0.9373 |
| INOV4 | 1411 | 0.9304 | 1013 | 0.9267 | 398 | 0.9374 |
| PROACT1 | 1411 | 0.9309 | 1013 | 0.9278 | 398 | 0.9369 |
| PROACT2 | 1411 | 0.9318 | 1013 | 0.9283 | 398 | 0.9385 |
| PROACT3 | 1411 | 0.9304 | 1013 | 0.9267 | 398 | 0.9374 |
| EUE1 | 1411 | 0.9321 | 1013 | 0.9281 | 398 | 0.9386 |
| EUE2 | 1411 | 0.9333 | 1013 | 0.9298 | 398 | 0.9399 |
| EUE3 | 1411 | 0.9338 | 1013 | 0.9302 | 398 | 0.9406 |
| EUE4 | 1411 | 0.9397 | 1013 | 0.9274 | 398 | 0.9370 |
| EUE5 | 1411 | 0.9317 | 1013 | 0.9282 | 398 | 0.9392 |
| Total scale |  | 0.9327 |  | 0.9293 |  | 0.9391 |

Source: Authors’ contribution in Stata 16

**Table S4.** Wald tests for equations associated with the SEM models

| SEM 1 | | | | SEM 2 | | | SEM 3 | | |
| --- | --- | --- | --- | --- | --- | --- | --- | --- | --- |
| Variables | Chi^2^ | df | *p*-value | Chi^2^ | df | *p*-value | Chi^2^ | df | *p*-value |
| EI1 | 0.00 | 0 | - | 0.00 | 0 | - | 0.00 | 0 | - |
| EI2 | 1346.91 | 1 | 0.000 | 866.64 | 1 | 0.000 | 480.66 | 1 | 0.000 |
| EI3 | 1718.79 | 1 | 0.000 | 1106.50 | 1 | 0.000 | 616.89 | 1 | 0.000 |
| EI4 | 2060.60 | 1 | 0.000 | 1307.65 | 1 | 0.000 | 769.91 | 1 | 0.000 |
| EI5 | 2083.55 | 1 | 0.000 | 1318.64 | 1 | 0.000 | 785.14 | 1 | 0.000 |
| EI6 | 2015.35 | 1 | 0.000 | 1281.41 | 1 | 0.000 | 747.83 | 1 | 0.000 |
| EI7 | 771.36 | 1 | 0.000 | 532.42 | 1 | 0.000 | 229.94 | 1 | 0.000 |
| EI8 | 699.03 | 1 | 0.000 | 434.73 | 1 | 0.000 | 266.84 | 1 | 0.000 |
| RISK1 | 0.00 | 0 | - | 0.00 | 0 | - | 0.00 | 0 | - |
| RISK2 | 577.82 | 1 | 0.000 | 384.80 | 1 | 0.000 | 168.62 | 1 | 0.000 |
| RISK3 | 939.92 | 1 | 0.000 | 624.17 | 1 | 0.000 | 273.69 | 1 | 0.000 |
| INOV1 | 0.00 | 0 | - | 0.00 | 0 | - | 0.00 | 0 | - |
| INOV2 | 480.61 | 1 | 0.000 | 337.02 | 1 | 0.000 | 136.76 | 1 | 0.000 |
| INOV3 | 453.76 | 1 | 0.000 | 322.61 | 1 | 0.000 | 125.81 | 1 | 0.000 |
| INOV4 | 466.90 | 1 | 0.000 | 330.40 | 1 | 0.000 | 130.49 | 1 | 0.000 |
| PROACT1 | 0.00 | 0 | - | 0.00 | 0 | 0.000 | 0.00 | 0 | 0.000 |
| PROACT2 | 357.02 | 1 | 0.000 | 246.39 | 1 | 0.000 | 107.88 | 1 | 0.000 |
| PROACT3 | 341.15 | 1 | 0.000 | 236.14 | 1 | 0.000 | 102.96 | 1 | 0.000 |
| EUE1 | 0.00 | 0 | - | 0.00 | 0 | 0.000 | 0.00 | 0 | 0.000 |
| EUE2 | 1986.51 | 1 | 0.000 | 908.27 | 1 | 0.000 | 446.47 | 1 | 0.000 |
| EUE3 | 1352.95 | 1 | 0.000 | 497.11 | 1 | 0.000 | 225.60 | 1 | 0.000 |
| EUE4 | 720.56 | 1 | 0.000 | 204.33 | 1 | 0.000 | 92.41 | 1 | 0.000 |
| EUE5 | 294.78 | 1 | 0.000 | 1392.13 | 1 | 0.000 | 599.49 | 1 | 0.000 |
| H0: All coefficients excluding the intercepts are 0.  We can thus reject the null hypothesis for each equation. | | | | | | | | | |

Source: Authors’ contribution in Stata 16

**Table S5.** Goodness-of-fit tests for the SEM models

|  | SEM 1 | SEM 2 | SEM 3 |
| --- | --- | --- | --- |
| “Likelihood ratio” | | | |
| “Model vs. saturated” chi^2^_ms (15) | 4065.014 | 2853.364 | 1453.106 |
| p > chi^2^ | 0.000 | 0.000 | 0.000 |
| “Baseline vs. saturated” chi^2^_bs (24) | 24717.708 | 17020.333 | 7918.951 |
| p > chi^2^ | 0.000 | 0.000 | 0.000 |
| “Information criteria” | | | |
| “AIC (Akaike's information criterion)” | 78934.320 | 56697.456 | 22256.199 |
| “BIC (Bayesian information criterion)” | 79322.972 | 57061.586 | 22551.196 |
| “Baseline comparison” | | | |
| “CFI (Comparative fit index)” | 0.843 | 0.843 | 0.840 |
| “TLI (Tucker–Lewis index)” | 0.824 | 0.824 | 0.820 |
| “Size of residuals” | | | |
| “SRMR (Standardized root mean squared residual)” | 0.025 | 0.014 | 0.051 |
| “CD (Coefficient of determination)” | 0.995 | 0.994 | 0.996 |

Source: Authors’ process in Stata 16
